# A Sub-Pixel Measurement Platform Using Twist-Angle Analysis in Two-Dimensional Planes

**DOI:** 10.3390/s25041081

**Published:** 2025-02-11

**Authors:** Jiangbo Lyu, Wenchao Kong, Yan Zhou, Yazhi Pi, Zizheng Cao

**Affiliations:** 1 Peng Cheng Laboratory, Shenzhen 518055, China; 2 Department of Electronic and Information Engineering, Harbin Institute of Technology (Shenzhen), Shenzhen 518055, China

**Keywords:** arrayed UV LED, measurement platform, twist-angle analysis, sub-pixel, spatial intensity distribution, response time

## Abstract

Arrayed ultraviolet (UV) LED light sources have been widely applied in various semiconductor processes, ranging from photopolymerization to lithography. In practical cases, based on data provided by manufacturers, calibration of individual UV LEDs is often needed before their real usage in high-precision applications. In this paper, we present a high-precision, automated light source measurement platform, which can be applied to the performance evaluation of various types of light sources. In order to minimize errors introduced by the automated measurement system, the platform employs a sub-pixel measurement technique, along with a twist-angle method, to perform multiple measurements and analyses of the spatial intensity distribution of the light source on a given plane. Through noise analysis of repeated measurements, the platform’s effectiveness and reliability are validated within a certain tolerance range. The high-precision automated light source measurement platform demonstrates excellent performance in the precise control and data acquisition of complex light sources. The light source dataset derived from the test results can provide guidance for the optimization of light sources in fields such as lighting, imaging, and lithography.

## 1. Introduction

With the rapid development of industrial technologies, advanced manufacturing techniques have been widely applied across various fields. Light source technology, particularly efficient and precise light sources, has become a core driving force behind these technological advancements [1,2,3]. As an emerging light source technology, arrayed UV LED light sources are gradually replacing traditional light sources due to their high luminous efficiency, long lifespan, superior controllability, and environmental friendliness [4,5,6]. They have found extensive applications in fields such as microelectronics manufacturing [7,8,9,10,11], photolithography [12,13,14], and curing processes [15]. However, in practical applications, the data provided by suppliers are not guaranteed to meet the requirements of high-precision applications directly. Consequently, calibration is usually necessary before utilizing UV LEDs for such purposes. The performance of light sources in these applications directly impacts process quality, system reliability, and overall efficiency [16,17]. Therefore, the precise characterization of light sources is crucial to ensure their optimal performance in practical applications.

However, existing light source testing platforms often face challenges, such as insufficient resolution and the inability to comprehensively capture critical parameters of the light source [18,19], including spatial intensity distribution and response time. The limitations of traditional platforms often result in measurements that lack precision or fail to reflect the actual performance of light sources in certain applications. Therefore, the development of a high-precision, highly automated testing platform has become a pressing technical challenge in the field of light source testing.

To address this issue, extensive research has been conducted on measurement techniques, and the application of super-resolution imaging has been explored to enhance the accuracy of light source measurements. As digital images serve as two-dimensional signal records, higher-resolution digital images are often required in many applications [5,20,21,22,23,24,25,26]. The need for resolution enhancement becomes particularly critical for the precise representation of the spatial intensity distribution of light sources [26,27,28,29,30]. Currently, there are two main approaches to achieving high-resolution images. The first involves reducing the pixel size [31,32,33,34]. However, as the pixel size decreases, new challenges arise, including a lower signal-to-noise ratio, increased pixel crosstalk, and reduced readout speed [35]. The second approach involves employing non-rectangular pixel layouts, increasing the camera focal length, or enlarging the chip size [36]. Yet, due to limitations in sensor technology and manufacturing processes, these methods are generally not considered effective solutions. Specifically, modern CCD and CMOS sensor manufacturing processes are primarily based on rectangular pixel layouts. Non-rectangular layouts introduce increased complexity in photolithography, etching, and alignment, resulting in higher defect rates and lower yields. Additionally, current sensor technology is predominantly optimized for rectangular arrays, making the effective production of non-rectangular layouts challenging [37,38].

Due to the limitations of hardware devices, super-resolution (SR) techniques are still required to achieve higher resolution. These techniques utilize signal processing methods to generate high-resolution (HR) images from multiple observed low-resolution (LR) images. The advantage of SR methods lies in their ability to overcome the limitations of LR observations without the cost to enhance hardware capabilities [39,40,41]. Given that light source measurement devices aim to capture spatial intensity distributions, a non-uniform interpolation approach is employed to reconstruct images and achieve super-resolution [34,42,43,44]. This method represents the most intuitive approach for SR image reconstruction and is widely employed in practice due to its broad applicability. By estimating relative motion information, an HR image is obtained at non-uniformly spaced sampling points. Subsequently, a uniformly spaced sampling grid is generated following either a direct or iterative reconstruction procedure. Gross [45] applied the generalized multi-channel sampling theorem introduced by Papoulis [46] and Brown [47] to perform non-uniform interpolation on spatially shifted LR images. This interpolation process was followed by a deblurring step under the assumption of precisely known relative displacements. Komatsu et al. [48] developed a method for obtaining high-resolution images from multiple simultaneous camera captures using the Landweber algorithm. They employed block-matching to measure relative motion. However, identical apertures in all cameras imposed strict constraints on their placement and scene configuration. This issue was mitigated by equipping the cameras with varying apertures. Hardie et al. [49] proposed a real-time infrared image registration and SR reconstruction technique, employing a gradient-based registration algorithm to estimate displacements and a weighted nearest-neighbor interpolation approach. Ultimately, Wiener filtering was used to suppress blur and system noise. Shah and Zakhor [29] adopted the Landweber algorithm for the SR enhancement of color video sequences, but to address registration inaccuracies, they evaluated multiple candidate motion estimates rather than a single motion vector per pixel, using both brightness and color information to compute the motion field. Nguyen and Milanfar [50] presented an efficient wavelet-based SR reconstruction algorithm. By exploiting the interlaced sampling grid inherent in SR, they derived a wavelet interpolation method tailored for interlaced two-dimensional data, reducing computation while maintaining reconstruction quality.

We propose a highly automated and high-precision light source testing platform. This platform employs a non-uniform interpolation method to integrate multiple low-resolution images, achieving high-resolution outputs enabling high-resolution outputs and precise characterization of spatial intensity distribution. Additionally, it is capable of measuring critical parameters of light sources, including response time. By implementing repeated measurement techniques, the platform effectively suppresses noise. The spatial intensity distribution obtained exhibits a Gaussian profile, confirming the platform’s effectiveness and reliability. The platform is not only applicable for testing ultraviolet LED array light sources but also capable of accurately measuring the key parameters of traditional laser light sources, making it suitable for performance evaluation across various types of light sources. The platform outputs test datasets for spatial intensity distribution and response time, providing essential data support for subsequent light source applications or optimizations. It offers significant potential for a wide range of applications.

## 2. Materials and Methods

### 2.1. Measurement Setup

**Figure 1:**
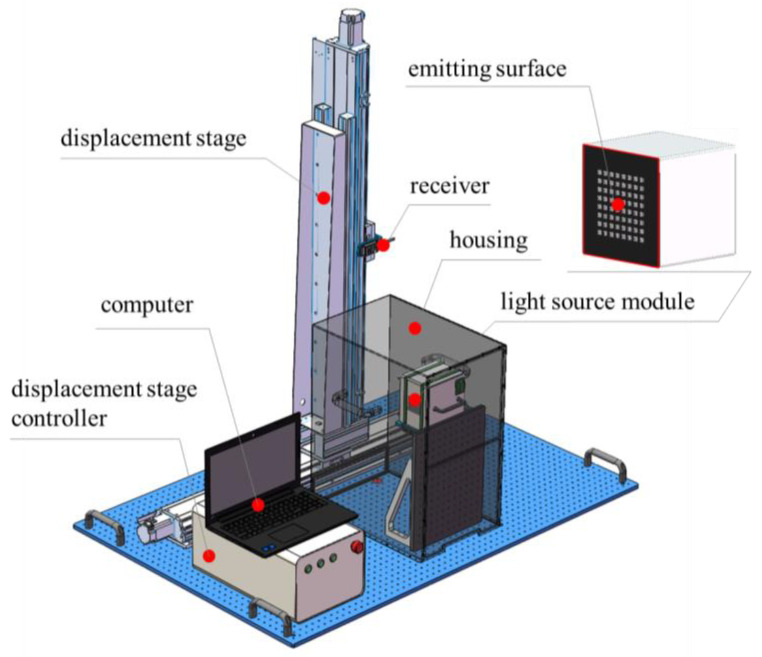
Schematic of the light source testing setup.

The structural schematic of the light source testing device is shown in Figure 1. The device adopts a vertical structural design to ensure the stable control of the relative position between the light source and the receiver.

The main components of the light source testing device include a displacement stage, a displacement stage controller (Wuhan RED STAR YANG TECHNOLOGY Co., Ltd., Wuhan, China), a receiver (UV radiometer or UV photodetector, Shenzhen Linshang Technology Co., Ltd., Shenzhen, China), a light source module, a housing, and a computer. The distance between the emitting surface of the light source module and the plane where the receiver is positioned is 100 mm. The displacement stage has a travel range of 1000 mm × 1000 mm, which is sufficient to cover the spatial intensity distribution range of most types of light sources. The UV radiometer enables high-sensitivity measurements, featuring an aperture diameter of 10 mm. The protective housing has dimensions of 335 mm × 620 mm × 500 mm, and the light source module measures 160 mm × 160 mm × 122 mm.

**Figure 2:**
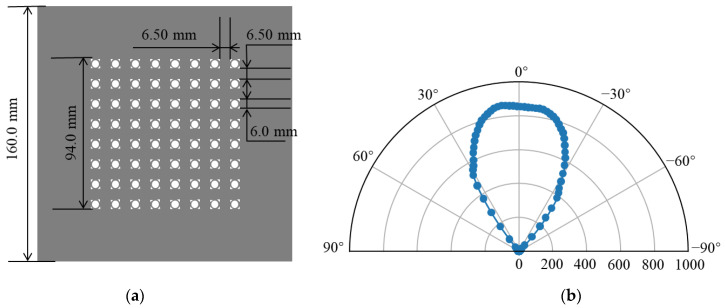
(**a**) Schematic of the UV LED array light source. (**b**) Polar luminous intensity distribution curve.

The light source is a UV array LED light source, mounted on a fixed bracket above the testing platform. Its structural schematic is shown in Figure 2. In Figure 2a, the light source device measures 160 mm × 160 mm, with the LED array occupying a central region of 94 mm × 94 mm. The UV array LED emits light with a central wavelength of 365 nm, and the array consists of 8 × 8 LEDs. Figure 2b shows the cross-sectional intensity distribution data provided by the supplier, obtained using an integrating sphere. In contrast, the objective of the light source measurement system is to capture the spatial intensity distribution in specific directions confined with a specific plan area, rendering a direct comparison between the two datasets impractical. According to the supplier’s data, the polar luminous intensity distribution conforms to the Lambertian source model. Therefore, we assume that the emission characteristics of the packaged LED adhere to the Lambertian source model [19], with its intensity given by Equation (1).

(1) $$I_{\varphi }=I_{0}\cdot {cos}^{m}\left(\varphi \right),$$

where φ denotes the viewing angle, $I_{0}$ represents the luminous intensity along the surface normal of the light source, $I_{0}$ is directly proportional to the irradiance, and m represents an important parameter describing the directional characteristics of the LED light source. It is determined by the LED’s half-power angle and is used to control the distribution of the LED light intensity at different angles.

These LEDs are evenly spaced, with a center-to-center distance of 12.5 mm between adjacent LEDs. The luminous intensity of the LEDs is controlled using pulse-width modulation (PWM) technology, with each LED offering 16 intensity levels. The central wavelength of the light source module is approximately 365 nm. The module employs an air-cooling system for heat dissipation, and the UV LED chip configuration employs the single-chip design, which not only possesses a low level of thermal effects but also significantly enhances the stability and reliability of the light source. The reduction in thermal effects subsequently enhances the stability and reliability of the light source, ensuring consistent performance and extended operational lifespan.

### 2.2. Testing Methods

**Figure 3:**
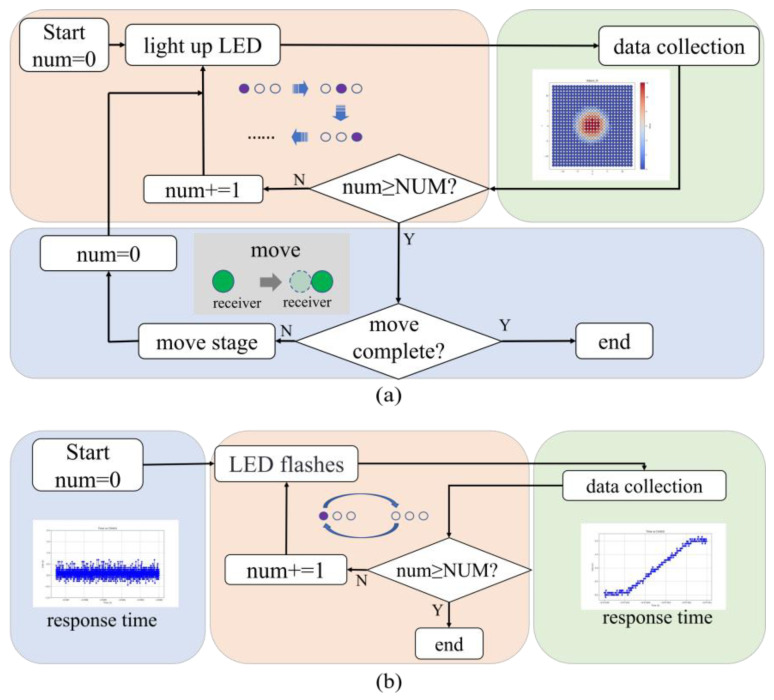
Workflow of the light source control and driving algorithm. (**a**) Spatial intensity distribution measurement process. (**b**) Temporal response measurement process.

Based on a standardized testing platform, a light source control driving algorithm has been developed. By coordinating light source control, displacement stage control, and receiver data acquisition, the algorithm enables the communication and control of various UV array LED light sources, movement of the two-dimensional displacement stage, and data reception and storage by the receiver. This achieves the measurement of the spatial intensity distribution and temporal response of the light source. The workflow of the light source control driving algorithm is shown in Figure 3. Figure 3a illustrates the testing process for spatial intensity distribution, where *num* denotes the LED index and *NUM* refers to the total number of light source LEDs (*NUM* = 64). In Figure 3a, the process consists of two nested loops: the inner loop switches the LEDs and collects data, while the outer loop updates the spatial position of the receiver and then repeats the inner loop. The UV radiometer, integrated with the two-dimensional translation stage, enables full control of the scanning path, allowing it to adapt flexibly to measurement areas of various shapes and sizes. A non-uniform interpolation method is employed in the testing process of spatial intensity distribution, as described by Equation (2).

(2) $$HR=\sum_{i}{LR}_{i},$$

where HR represents the high-resolution image, LR denotes the low-resolution image, and i indicates the index of the low-resolution image sequence. This method leverages a single-pixel information acquisition approach, combining the illumination area of the light source with a radiometer featuring a circular aperture. By utilizing a two-dimensional displacement stage to scan the entire plane point by point, a complete image frame can be generated upon the completion of the scan. This method allows for the collection of multiple low-resolution image frames within the testing area, which can then be used to reconstruct high-resolution images, achieving super-resolution.

Figure 3b illustrates the response time testing process. The UV photodetector measured the transient time response of the UV array LED light source through its flicker behavior.

**Figure 4:**
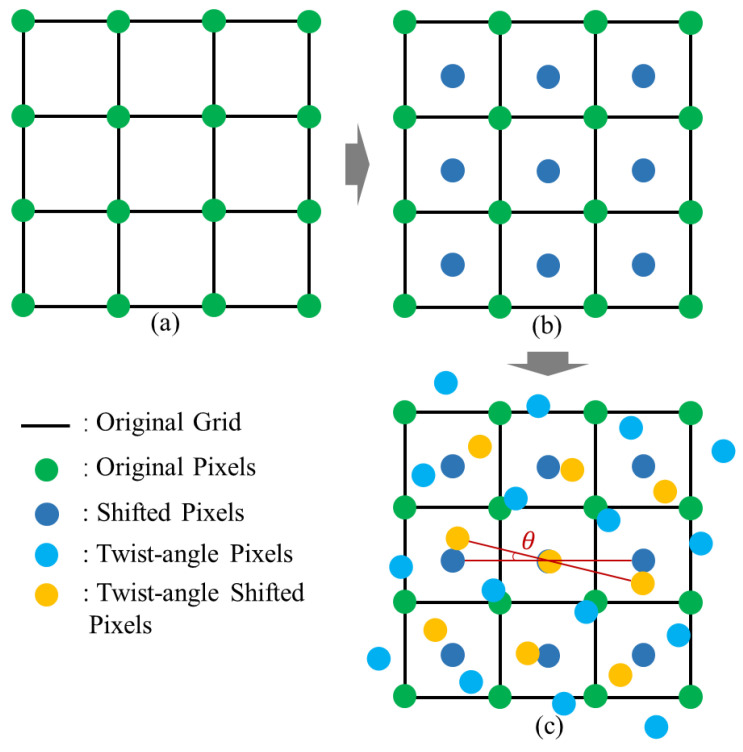
Schematic diagram of measurement method. (**a**) Grid sampling. (**b**) Sub-pixel sampling. (**c**) Multi-frame superposition.

The sampling point distribution following the process outlined in Figure 3a is shown in Figure 4a. The black lines represent the grid of the scanning area, while the green circles indicate the positions where the UV radiometer collected spatial intensity distribution data. These collected data are considered a single image ${LR}_{1}$, arranged in a grid pattern. The grid length was 10 mm, indicating that the resolution of this image was 10 mm.

To further enhance the resolution, sub-pixel precision shifting and sampling [51] were performed based on the data collected in Figure 4a. The resulting schematic is shown in Figure 4b. The green filled circles represent the first image frame collected in Figure 4a, while the blue circles correspond to the second image frame ${LR}_{2}$ obtained through sub-pixel sampling. Since the collected data were sampled based on spatial coordinates, the two frames of data could be directly integrated into a reconstruction image using a sub-pixel interpolation method. The reconstructed image demonstrates a significantly improved resolution compared to the original.

Based on the detachable convenience of the light source module and the precise positioning control of the two-dimensional displacement stage, it is important to emphasize here that light source module rotation has been introduced in addition to the aforementioned two image frames (${LR}_{3}$ and ${LR}_{4}$). The rotation was achieved by adjusting the height of the carrier stage and placing a precision-machined calibration block beneath the light source, both with micron-level accuracy, ensuring controlled and precise rotation. This involved a relative rotation of the data acquisition plane, enabling the collection of two additional image frames: the grid sampling and sub-pixel sampling data after a rotation of angle θ. The schematic of these four frames is shown in Figure 4c. By reconstructing the image from these four low-resolution frames using a non-uniform interpolation method, super-resolution imaging HR was achieved.

By repeatedly measuring the spatial intensity distribution within the testing area, the impact of noise on the results was significantly reduced, thereby improving the signal-to-noise ratio and significantly enhancing the reliability of the testing outcomes. Compared to pixel arrays, this approach greatly reduces the hardware complexity and substantially simplifies costs.

## 3. Results and Discussion

The light source testing apparatus is capable of measuring key parameters of the light source, including the spatial intensity distribution, temporal response, and other critical characteristics.

### 3.1. Spatial Intensity Distribution

**Figure 5:**
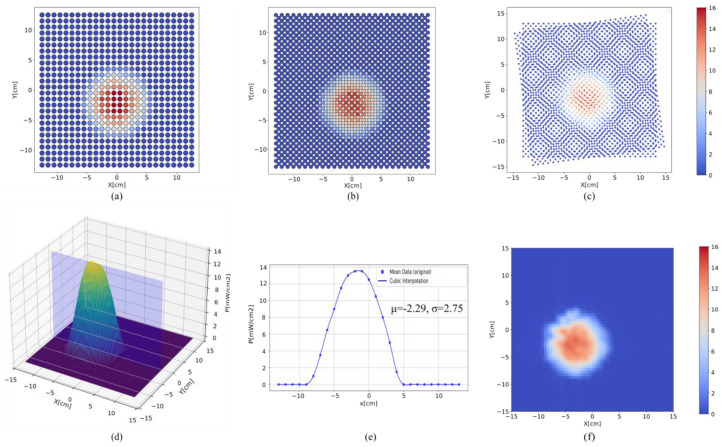
Spatial intensity distribution results. (**a**) Grid sampling. (**b**) Sub-pixel sampling. (**c**) Multi-frame superposition. (**d**) Three-dimensional irradiance data distribution. (**e**) Irradiance data distribution of a specific cross-section. (**f**) Super-resolution reconstruction analyzed using the Fourier method.

During the grid data acquisition process, the measurement area was set to 260 mm × 260 mm, and the LED intensity level was configured to level 16. The first frame ${LR}_{1}$ measurement results of a specific LED are shown in Figure 5a. The total measurement time depends on the area to be scanned on the receiver’s surface. For each pixel, the data acquisition time was set to 3 s, while the displacement stage required 2 s to move 1 cm. Given that the LED array consisted of 64 light sources and the measurement grid comprised 27 × 27 pixels, the estimated time to complete a full measurement cycle for one frame was approximately 39 h. Figure 5a illustrates the spatial intensity distribution of the LED in the array, with the results represented as a two-dimensional grid. The horizontal and vertical axes represent the spatial coordinates in the x- and y-directions, respectively. The color bar on the right ranges from blue to deep red, indicating irradiance values from low to high within the two-dimensional spatial region. This visual representation uses color to depict the numerical values of the spatial intensity distribution of light. The spot diameter was approximately 100 mm, from which the beam angle of the LED was determined to be 60°.

The second frame image ${LR}_{2}$ was obtained using sub-pixel sampling for data acquisition. Although the resolution of the equipment itself was limited, sub-pixel sampling captured the details between pixels, effectively simulating smaller spatial units. This approach enhanced measurement accuracy without increasing the actual hardware sampling costs. The test results are shown in Figure 5b. By utilizing sub-pixel precision shifting, the second frame image captured data from the remaining blank regions. To avoid image distortion, the pixel points are refined in Figure 5b, thereby enhancing the visual effect. Compared to Figure 5a, the points in Figure 5b are more densely distributed within the same spatial coordinates. Specifically, the number of pixels increased from 27 × 27 to 27 × 27 + 26 × 26, thereby improving the resolution.

To more accurately reproduce the distribution of the light source from different perspectives, the rotation of the measurement plane was introduced. When collecting the third and fourth frames (${LR}_{3}$ and ${LR}_{4}$), the light source was rotated by 8°, causing the pixel points originally aligned in the same row or column to no longer overlap. At different spatial positions, the angles between the UV radiometer and the LEDs varied, allowing for the acquisition of more independent and non-interfering measurement points even within the same scanning area. This effectively increased the resolution of the data acquisition. Rotating the light source by 8° is equivalent to a −8° rotation of the testing plane. During data visualization, the data collected for the third and fourth frames must be rotated and combined with the first two frames using a non-uniform interpolation method to reconstruct a new image HR. The reconstructed image can achieve sub-micron resolution. The results are shown in Figure 5c. By stacking multiple frames of images, the integrity of the data was ensured, and the results are more closely aligned with the true distribution. The rotation matrix is provided in Equation (3), and the coordinate transformation formula is shown in Equation (4).

(3) $$\left[\begin{array}{cc} cos\left(\theta \right) & -sin\left(\theta \right)\\ sin\left(\theta \right) & cos\left(\theta \right) \end{array}\right],$$

(4) $$\left[\begin{array}{c} x^{\prime }\\ y^{\prime } \end{array}\right]=\left[\begin{array}{cc} cos\left(\theta \right) & -sin\left(\theta \right)\\ sin\left(\theta \right) & cos\left(\theta \right) \end{array}\right]\left[\begin{array}{c} x-x_{c}\\ y-y_{c} \end{array}\right]+\left[\begin{array}{c} x_{c}\\ y_{c} \end{array}\right],$$

where ($x^{\prime }$, $y^{\prime }$) are the new coordinates after rotation, (x, y) are the original coordinates of each point, and ($x_{c}$, $y_{c}$) is the center of rotation.

To verify whether the repeated measurement results follow a Gaussian distribution, the mean of the three measured datasets was calculated, followed by interpolation to generate a 3D plot, as shown in Figure 5d. Figure 5d depicts the 3D data distribution, while Figure 5e shows the data distribution of a specific cross-section. Ideally, the irradiance density distribution should exhibit a Gaussian profile, as described by Equation (5).

(5) $$P\left(x\right)=P_{0}exp\left(-\frac{{\left(x-\mu \right)}^{2}}{{2\sigma }^{2}}\right)$$

where $P_{0}$ represents the peak intensity, x denotes the position, μ is the mean, and σ refers to the standard deviation.

The cross-sectional data distribution was further analyzed by calculating its mean and standard deviation, with the corresponding formulas given in Equations (6) and (7). The resulting mean and standard deviation were 2.29 and 2.75, respectively. After calculating the mean and standard deviation of the sampled data, all measured values were found to lie within the μ(x) ± 3σ(x) range. Considering that this range encompasses approximately 99.73% of random fluctuations under an assumed normal distribution, these results indicate that the data variability in this study fell within an acceptable range and thus demonstrates a high level of overall reliability. This finding not only confirms the conformity of the light source to the Lambertian model but also, from a statistical perspective, demonstrates the high repeatability of the measurement system.

(6) $$\mu \left(x\right)=\frac{1}{n}\sum_{i=1}^{n}P_{i}\left(x\right)$$

(7) $$\sigma \left(x\right)=\sqrt{\frac{1}{n}\sum_{i=1}^{n}{\left(P_{i}\left(x\right)-\mu \left(x\right)\right)}^{2}}$$

The super-resolution results were analyzed using the Fourier method, as shown in Figure 5f. The spatial light intensity distribution exhibits a pronounced central symmetry and generally aligns with the characteristics of a Gaussian distribution. The intensity is highest at the center and gradually decreases as the spatial distance increases. This indicates a high degree of energy concentration in the light source, and the spatial distribution matches theoretical expectations. By performing multiple repeated measurements and averaging the results, the impact of random noise was significantly reduced, effectively suppressing background noise levels and accentuating the signals’ primary peak characteristics. The Fourier transform results indicate that the light intensity was predominantly concentrated in low-frequency components, further confirming the effectiveness and reliability of the light source testing apparatus. These findings serve as an important reference for evaluating light source performance and optimizing the experimental setup.

In addition, we compared the performance of different platforms, including resolution and key light source parameters, as shown in Table 1. The comparison results indicate that the proposed platform is capable of measuring a more comprehensive set of light source characteristics, thus enabling a more thorough evaluation of the light source properties.

**Figure 6:**
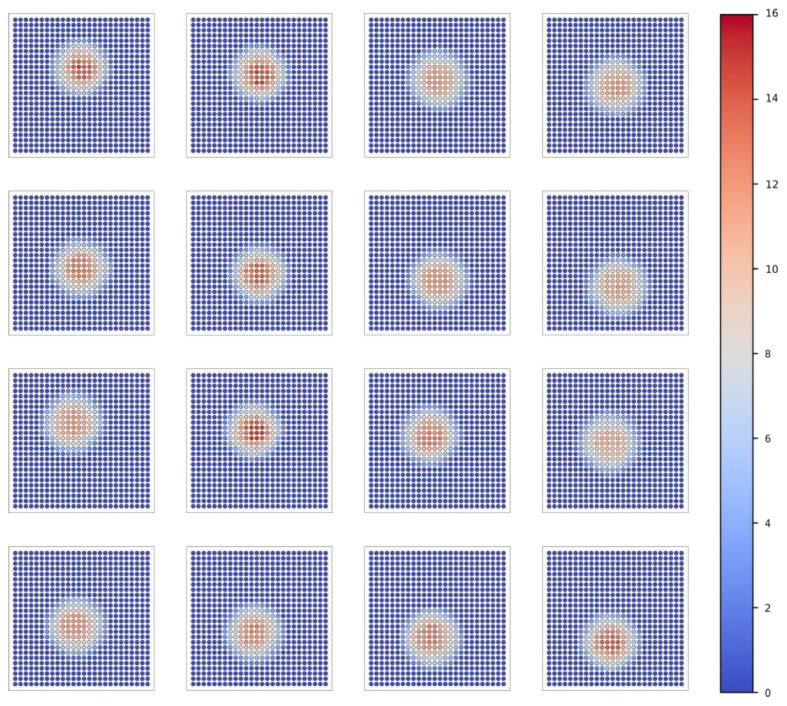
Grid sampling spatial intensity distribution results of 16 LEDs.

**Figure 7:**
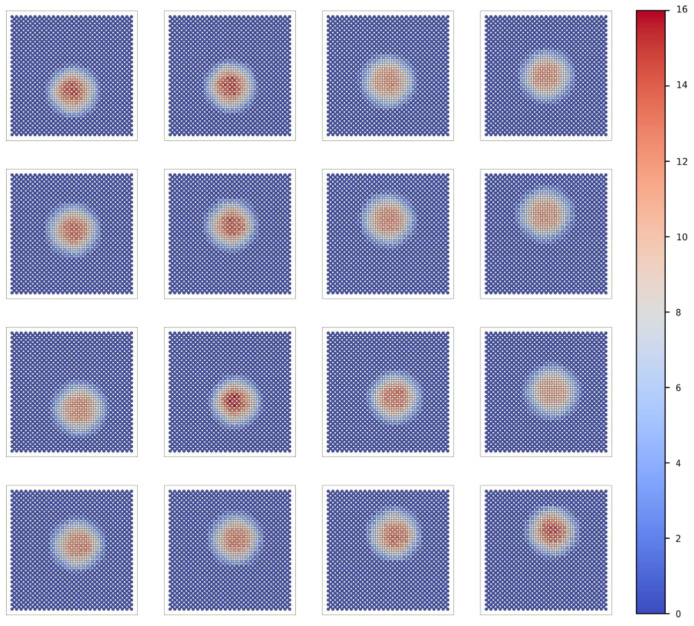
Sub-pixel sampling spatial intensity distribution results of 16 LEDs.

**Figure 8:**
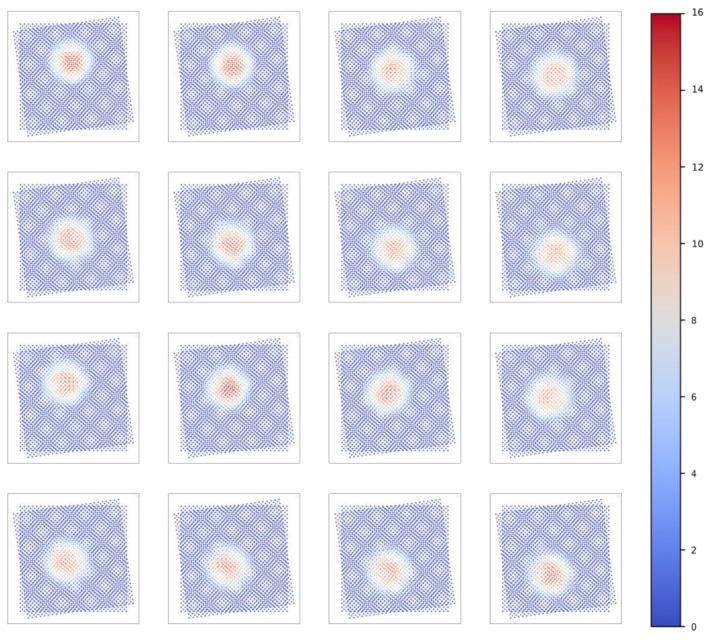
Multi-frame superposition spatial intensity distribution results of 16 LEDs.

**Figure 9:**
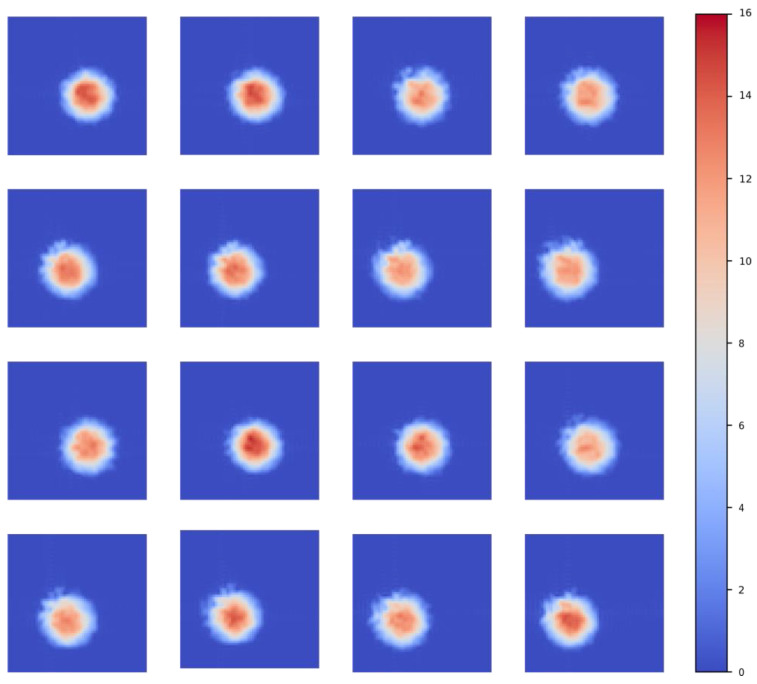
Super-resolution reconstruction results of 16 LEDs analyzed using the Fourier method.

Based on the aforementioned analysis methods, in addition to the results of a single LED, we also provide the test results for the remaining 15 LEDs. The data are visualized and represented through images, thereby emphasizing the reliability and versatility of the light source testing system. Figure 6 presents the spatial intensity distribution of 16 LEDs under grid sampling. Figure 7 illustrates the spatial intensity distribution of 16 LEDs under sub-pixel sampling. Figure 8 depicts the spatial intensity distribution of 16 LEDs obtained from angular measurements. Figure 9 analyzes the super-resolution reconstruction results of 16 LEDs using the Fourier method.

### 3.2. Response Time Testing

When testing the response time, the receiver needed to be replaced with a UV photodetector, which was fixed within the range illuminated by the LED under test. The UV photodetector had a minimum response time of 2 µs and was connected to the KEYSIGHT DSOX1204A oscilloscope. Additionally, the LED under testing was set to blink at a frequency of $10^{6}$ Hz, allowing the test results to be saved and analyzed via the oscilloscope. The parameters of the oscilloscope used during the measurement were as follows: 4 channels, a 600 MHz bandwidth, and a 5G Sa/s sampling rate.

**Figure 10:**
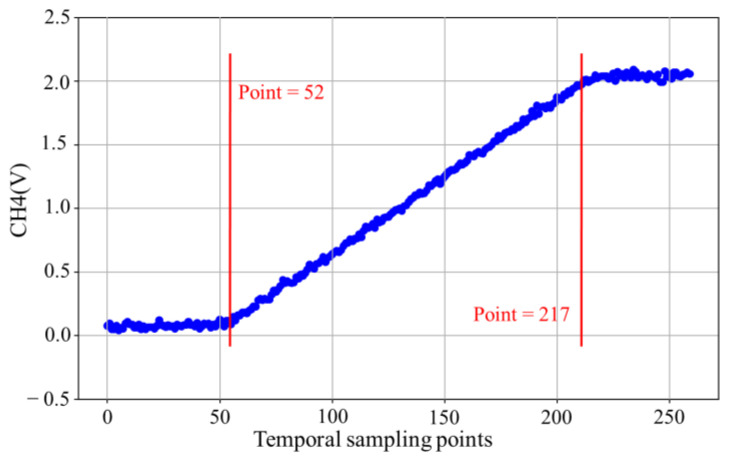
The rising edge of the response time waveform.

The response time under investigation refers to the transient response of the light source, specifically the rising or falling edge. Figure 10 presents the averaged rising edge results of 16 LEDs, where the horizontal axis represents the sampling points recorded by the oscilloscope, and the vertical axis indicates the averaged voltage levels of the 16 LEDs. A total of 165 points were identified within the voltage range of 0 V to 2 V. Given that the oscillator’s sampling rate was 5 GSa/s, each point corresponds to 0.0002 μs. Therefore, the 165 points on the rising edge represent a rise time of 33 μs.

In addition, using this light source testing apparatus, it is possible to collect data on various spatial positions of an 8 × 8 UV array LED light source at an intensity level of 16. This yields a rich training dataset, which can be utilized for further improvements and practical applications of the light source.

## 4. Conclusions

A highly automated and precise light source testing platform has been developed to measure the key parameters of light sources, such as the spatial intensity distribution and temporal response. By employing advanced methodologies, such as sub-pixel displacement and rotation angle adjustments, combined with state-of-the-art techniques, like sub-pixel sampling and non-uniform interpolation, the platform effectively reconstructs low-resolution images into high-resolution representations. This process ensures the accurate characterization of critical light source properties, enabling the precise evaluation and analysis of their performance metrics.

The spatial intensity distribution results demonstrate that the light source exhibited Gaussian characteristics, with high energy concentration and strong theoretical consistency. The temporal response measurements reveal the transient time response of the light source, providing indispensable information for high-precision applications. Additionally, Fourier-based reconstruction improved the measurement resolution, while noise reduction through repeated measurements enhanced the accuracy, further validating the effectiveness and reliability of the light source testing platform. These advancements lay a solid foundation for the continued development of light source design and evaluation.

Furthermore, the platform can perform comprehensive spatial and temporal data acquisition for an array of UV LEDs across 16 intensity levels, thereby generating a rich training dataset that can provide valuable opportunities for light source optimization and broader applications.

## Figures and Tables

**Table 1:** The performance comparison among different measurement platforms.

Methods	Resolution	Spatial Intensity Distribution	Response Time
Subpixel detector shift [52]	~200 μm	√	×
Holographic pixel super-resolution [53]	<1 μm	√	×
This work	<1 μm	√	√

## Data Availability

The data presented in this study are available upon request from the corresponding author. The data are not publicly available due to privacy.
